# Advanced pathophysiology mimicking lung models for accelerated drug discovery

**DOI:** 10.1186/s40824-023-00366-x

**Published:** 2023-04-26

**Authors:** Thanh Huyen Phan, Huaikai Shi, Christopher E. Denes, Alexander J. Cole, Yiwei Wang, Yuen Yee Cheng, Daniel Hesselson, Susan H. Roelofs, Graham Gregory Neely, Jun-Hyeog Jang, Wojciech Chrzanowski

**Affiliations:** 1 The University of Sydney, Sydney Nano Institute, Faculty of Medicine and Health, Sydney School of Pharmacy, Pharmacy and Bank Building A15, Camperdown, NSW 2006 Australia; 2 Burns Research and Reconstructive Surgery, ANZAC Research Institute, Concord Hospital, University of Sydney, Sydney, Australia; 3 Asbestos Disease Research Institute, Concord Hospital, Sydney, Australia; 4 The Dr. John and Anne Chong Lab for Functional Genomics, Charles Perkins Centre and School of Life & Environmental Sciences, The University of Sydney, Sydney, NSW 2006 Australia; 5 Centenary Institute, The University of Sydney, Sydney, NSW 2006 Australia; 6 Faculty of Medicine and Health, The University of Sydney, Sydney, NSW 2006 Australia; 7 Jiangsu Provincial Engineering Research Centre of TCM External Medication Development and Application, Nanjing University of Chinese Medicine, Nanjing, China; 8 Institute for Biomedical Materials and Devices (IBMD), School of Mathematical and Physical Sciences, Faculty of Science, University of Technology Sydney, Broadway, NSW 2007 Australia; 9 Locsense B.V., Locsense B.V., Enschede, The Netherlands; 10 Department of Biochemistry, College of Medicine, Inha University, Incheon, 400-712 South Korea

**Keywords:** Lung-mimicking models, Multimodal characterisation, Physiological relevance, Microcirculation, Extracellular matrix, Patient-derived cell lines, Personalised medicine

## Abstract

**Background:**

Respiratory diseases are the 2*nd* leading cause of death globally. The current treatments for chronic lung diseases are only supportive. Very few new classes of therapeutics have been introduced for lung diseases in the last 40 years, due to the lack of reliable lung models that enable rapid, cost-effective, and high-throughput testing. To accelerate the development of new therapeutics for lung diseases, we established two classes of lung-mimicking models: (i) healthy, and (ii) diseased lungs – COPD.

**Methods:**

To establish models that mimic the lung complexity to different extents, we used five design components: (i) cell type, (ii) membrane structure/constitution, (iii) environmental conditions, (iv) cellular arrangement, (v) substrate, matrix structure and composition. To determine whether the lung models are reproducible and reliable, we developed a quality control (QC) strategy, which integrated the real-time and end-point quantitative and qualitative measurements of cellular barrier function, permeability, tight junctions, tissue structure, tissue composition, and cytokine secretion.

**Results:**

The healthy model is characterised by (i) continuous tight junctions, (ii) physiological cellular barrier function, (iii) a full thickness epithelium composed of multiple cell layers, and (iv) the presence of ciliated cells and goblet cells. Meanwhile, the disease model emulates human COPD disease: (i) dysfunctional cellular barrier function, (ii) depletion of ciliated cells, and (ii) overproduction of goblet cells. The models developed here have multiple competitive advantages when compared with existing in vitro lung models: (i) the macroscale enables multimodal and correlative characterisation of the same model system, (ii) the use of cells derived from patients that enables the creation of individual models for each patient for personalised medicine, (iii) the use of an extracellular matrix proteins interface, which promotes physiological cell adhesion and differentiation, (iv) media microcirculation that mimics the dynamic conditions in human lungs.

**Conclusion:**

Our model can be utilised to test safety, efficacy, and superiority of new therapeutics as well as to test toxicity and injury induced by inhaled pollution or pathogens. It is envisaged that these models can also be used to test the protective function of new therapeutics for high-risk patients or workers exposed to occupational hazards.

**Graphical Abstract:**

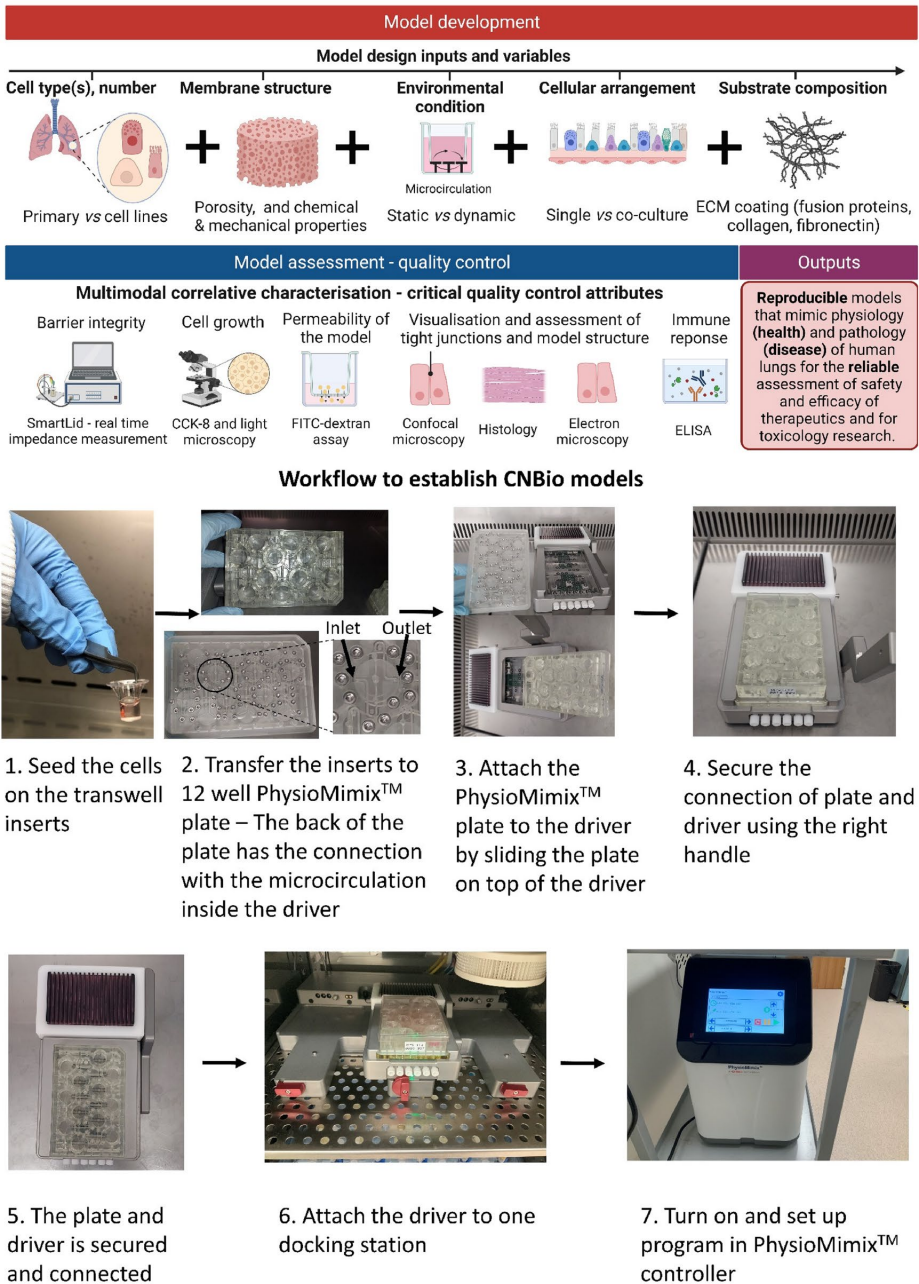

**Supplementary Information:**

The online version contains supplementary material available at 10.1186/s40824-023-00366-x.

## Introduction

Lung diseases are a leading cause of death and morbidity worldwide [1]. Chronic obstructive pulmonary disease (COPD, emphysema), which was an underlying cause of 3.23 million deaths in 2019 [2], is the most prevalent lung health complication globally [3]. According to the World Health Organisation, COPD incidence will more than double by 2030 and these numbers are expected to increase even more dramatically with worsening air quality and the COVID-19 crisis [4]. COPD leads to small airway obstruction and emphysema, which result in lung tissue damage, a persistent demand for medication and a reduced quality of life [5]. The damage in lungs caused by COPD cannot be reversed using current therapeutic strategies, which are only supportive, and do not promote tissue regeneration [6]. Thus, COPD is incurable. Innovative approaches such as stem cell-based regenerative medicines have shown a potential to repair and/or prevent lung deterioration [7]. However, these approaches have not been shown to be effective in clinical trials for the treatment of COPD [7]. Consequently, novel therapeutics for lung diseases are critically needed. To accelerate their clinical translation, we must first establish lung pathophysiology models that enable rapid, reliable, and reproducible testing of their safety and efficacy. These models will not only speed up the discovery of new strategies to repair lungs, but they will also provide a substantially greater confidence on their safety and superiority.

Traditionally, the development and assessment of new therapeutics for COPD utilise animal models. However, the anatomy, immune system, and inflammatory responses of animal lungs differ substantially from those in humans [8]. In addition, many animal models do not allow researchers to utilise the same inhalation/aerosol devices to deliver therapeutics as those used for humans. The delivery method is one of the key determinants of the treatment effectiveness (drug delivery to lungs); without a suitable method of delivering to lungs or where they are needed in the body, their real-world applications are limited [9, 10]. In addition, several studies of drug efficacy showed limited correlation between animals and human trials [10, 11].

Therefore, developing alternatives to animal models is one of the priorities in biotechnology, toxicology, pharmaceutical and medical research. Academia and industry have made tremendous progress on this front, including organ on a chip, organoids, and 3D bioprinted organ-like structures. For example, lung-on-chip microfluidics with a smoke generator and a micro-respirator overcame some of the limitations of animal models including ethics and cost [12]. However, these animal model alternatives also have significant shortcomings: for example, it is difficult to perform multimodal characterisation of a single model due to its small size (submillimeter), and thus it is problematic to validate such models which may negatively impact their reliability. Microfluidics house a limited number of cells and limited cellular crosstalk, which prevent cell proliferation and the growth of tissue as in human lungs. Efforts have been made to overcome these problems such as the uses of transwells to establish larger scale 3D lung models [13]. These models are maintained in static culture conditions, which means that the dynamic nature of human lungs/body and disease are not mimicked appropriately and it is difficult to interrogate real time cellular responses in these systems [13]. In summary, current in vitro lung models failed to reproduce the complexity of human lung pathophysiology, owing to the absence of integration between lung-mimics (both physiological and pathophysiological) and microenvironment (fluid perfusion, air liquid interface, cell–cell interaction).

### Concept development

Here, we designed, manufactured and validated advanced in vitro lung models that emulate human patho/physiology more accurately by implementing innovative biodesign elements: (i) microcirculation that maintains the physiological microenvironmental conditions, (ii) use of extracellular matrix peptides for substrate functionalisation, to promote the desired patho/physiological architecture and biomechanical properties of the tissue models, and (iii) use of patient-derived and relevant cells that enable the establishment of lung models for personalised medicine.

**Fig. 1:**
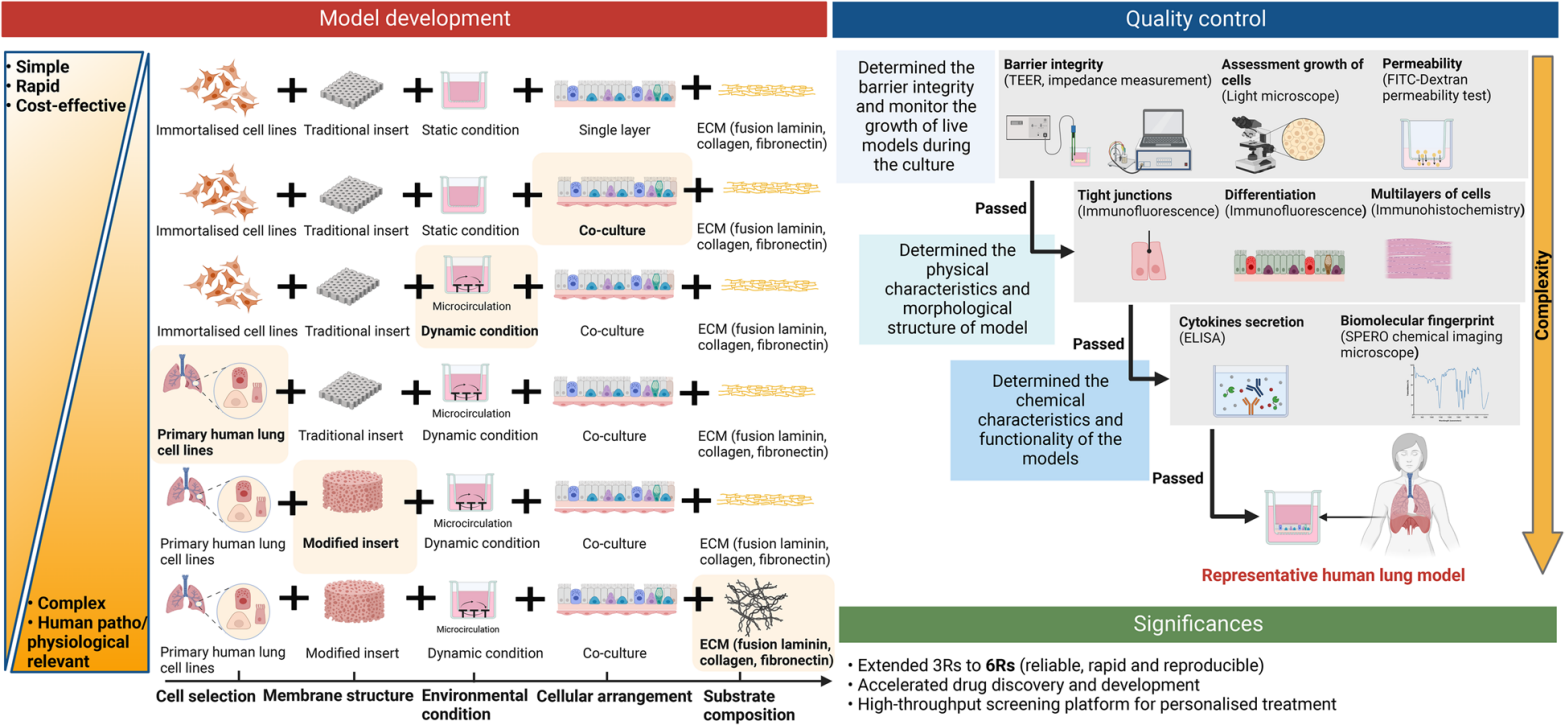
Overall scheme showing five key elements in model development, quality control of the models using multimodal characterisation and the significances of the developed models

The aim of this study was to investigate how these innovative elements affect the performance and functionality of the models. When developing a lung model, it is essential to identify physiological parameters that are relevant to a specific research question that can be addressed using the model. For example, if the aim is to screen compounds and determine their effect on the barrier function or permeability through the barrier, then 2D monolayer model and the measurements of transepithelial resistance and/or permeability assay might be sufficient and economically justified. However, if we want to gain a mechanistic understanding of how a specific compound drives tissue regeneration and recovery of lung function, then the model must emulate 3D complexity of tissue and the dynamic nature of the lung microenvironment *e.g.,* microcirculation. Therefore, we provide for the first time the comprehensive comparison between different models with varying complexity that can be used for particular applications/studies. To conceptualise, establish, and refine the in vitro lung model we considered five design components: (i) cell type, (ii) membrane structure/constitution, (iii) environmental conditions, (iv) cellular arrangement, (v) substrate, matrix structure and composition (Fig. 1).

To determine whether the lung models are reproducible and reliable it is necessary to develop bespoke quality control (QC) measures. QC also enables monitoring and assessing the functionality of the models during treatment. Hence, to establish the QC, we integrated the real-time and end-point quantitative and qualitative measurements of cellular barrier function, permeability, tight junctions, tissue structure, tissue composition, and cytokine secretion. The advantage of our models is that QC measurements can be performed simultaneously for each individual model in real time, which is not trivial, and applicable for traditional animal in vivo models. Applying the QC criteria, our models not only satisfy the current 3Rs (replacement, reduction, and refinement) in the animal uses, but they extend 3Rs to 6Rs through achieving reliability, robustness, and reproducibility. Notably, the comprehensive QC assessment of the models enabled us to establish and validate a rapid and streamlined QC protocol using a real-time, single bioimpedance measurement. We showed that bioimpedance was rapid (enabling high-throughput readout) and reliable, providing both qualitative and quantitative results. This technological innovation will lead to substantial labour- and cost-reduction and improve the relationship between the high complexity and low throughput of the models [14].

### Significances

This work is significant because it developed the alternative models that can potentially replace animals in research and accelerated the development of personalised medicine. Additionally, it is likely to increase the confidence on the safety, efficacy and superiority of new drugs and therapies. Furthermore, this work provided cost-effective and reliable assessment of new therapeutics for lung disease COPD, as well as assessment of the effects of pollutions on lungs and development of preventive therapies. This study will reduce the occurrence of development of lung injury, providing a healthier and sustainable future.

## Materials and methods

### Development of the model

#### Selection of cell types

The choice of cells is the most important determinant of any in vitro model. For the initial optimisation of the model fabrication and establishing quality control protocols, we used two immortalised cell lines [15, 16]: bronchial epithelial (BEAS-2B) and lung cancer cell line (A549). Cell lines are considered more reproducible, and relatively easier to culture and maintain. The use of the cell lines enabled us to refine our protocols, and as a result, accelerated establishing models that utilised primary cells. Models based on cell lines could also be considered as alternative models for research where the use of primary cells is not justified (e.g., due to the cost), and ‘simpler’ models can be used. The mono- and co-culture of the two cell lines (BEAS-2B and A549) were used in our study. The use of one cell line allowed us to examine the structure of the model and understand the limitations in monoculture systems *e.g.,* relatively poor barrier integrity. The use of two cell lines enabled us not only to determine the feasibility of co-culturing the cells derived from different parts of the lungs – a prerequisite to establishing co-culture models – but also to account for the intercellular communication, which regulates many of the biological processes e.g., protein production, tissue regeneration. The cell-to-cell communication is known to influence cell behaviour and function; hence the use of different cell types that co-exist in the human lungs is likely to alter the functionality of the models, which includes modulation of barrier function, the formation of polarized cells and tight junctions. Therefore, to determine the effects of intercellular communication on the model development, we use the above-mentioned cell lines and hypothesised that the use of two cell lines will allow us to mimic in vivo conditions more closely and establish more physiologically relevant lung models. Indeed, BEAS-2B and A549 have been used intensively to investigate the potency and safety of different materials [17, 18]. BEAS-2B (ATCC: CRL-9609) and A549 (ATCC: CCL-185) were cultured and maintained in Dulbecco's Modified Eagle Medium (DMEM medium–high glucose, Sigma-Aldrich, Australia).

To create a complex model that is more representative of human lungs, we selected two primary human cell lines – normal human bronchial epithelial (NHBE) and normal human lung fibroblasts (NHLF). NHLFs were cultured and maintained in Fibroblast growth medium-2 (FGM-2, Lonza, USA). Primary NHBE and diseased human bronchial/tracheal epithelial cells—COPD (DHBE) were cultured and maintained in Bronchial epithelial growth medium (BEGM, Lonza, USA). PneumaCult™-ALI medium (STEMCELL Technologies™, Australia) was used during the last three weeks of differentiation of the models in air liquid interface (ALI) condition (PneumaCult™-ALI medium was used in basolateral compartment and no medium was used in the apical compartment).

#### Modification of transwell inserts with different pore-density to grow the culture

The pore-density of the insert membranes used for culture was shown to influence the proliferation and differentiation of the models grown at an ALI [19]. Therefore, to investigate the optimal pore-density of insert membranes for culture, two common insert types with different pore-densities were used. These inserts are: (i) available standard Corning with 4 × 10^6^ pores/cm^2^ (Cat: COR3470, Corning, Life Sciences), and (ii) CellQART with 100 × 10^6^ pores/cm^2^ (Cat: 9,320,402, Sabeu, Germany). Both inserts were cultured and maintained under the same conditions: seven days in the liquid phase and three weeks in the ALI phase. The medium of these culture was changed every two days and the impedance measurement (Locsense, Netherland) was performed every two days to monitor the growth of the model.

#### Topographical and nanomechanical characterization of the membranes

To examine topography, pore size and distribution as well as to probe the nanomechanical properties of two membrane inserts, Atomic Force Microscopy was used (AFM, Asylum Research, USA). Force-volume mode was applied and a silicon nitride cantilever with a nominal spring constant of 0.409 N/m (HYDRA-ALL-G-50, AppNano, CA, USA) was used. Membrane insert was attached to a petri dish (Bacteriological petri dish, Falcon®, Corning, USA) and submerged in PBS. The contact mode was used for topographic imaging and the scan rate for topography was 0.5 Hz. To determine the stiffness distribution of the Corning insert (stiffness map), 800 points were indented for each scan area (25 × 50 µm) and a minimum of 5 areas was required for each sample. The force-distance curve of the membrane insert was constructed based on the deflection of the cantilever and the displacement in the z-direction. The individual curves were used to calculate apparent elastic modulus and then used to generate stiffness maps. The stiffness maps represent the distribution of the apparent elastic modulus of the membrane surface. For the CellQart membrane, the stiffness map cannot be automatically generated due to the presence of large number of pores and the indentation in the porous area will skew up the data and present inaccurate stiffness distribution. To overcome this problem, we recorded individual indentation curves in the areas between the pores as identified in the topography images. For each sample, we recorded a minimum of 40 individual force-distance curves from the 10 × 20 µm scan area. Apparent elastic modules were calculated from each individual curve. To analyse the difference in the stiffness between two membrane inserts, linear regression analysis based on log normal stiffness and a cumulative frequency was performed on each histogram.

#### Optimisation of the environmental condition using microfluidic 3D system

Dynamic condition is one of the key parameters to establish a physiologically relevant model. To mimic the dynamic flow condition in human lungs, microfluidic 3D system was created using CNBio’s PhysioMimix™ (CNBio Innovations, Welwyn Garden City, Hertfordshire, UK). Specifically, cultures were performed in the multi-MPS12 plate (MPS-12) with the media flow in the basal compartment at a flow rate of 0.5 µL/s. The multi-MPS12 plate was primed with water on Day 1 and cell culture medium on Day 2 before commencement of cell culture. Cells were maintained in one direction flow for the whole duration of the culture. Cell culture media was replaced every 48 h.

#### Optimisation of the cellular arrangement of the models

To represent the cellular arrangement thoroughly by creating cell–cell interactions and enhancing the reliability of the models, co-cultures of two cell lines were performed. Mono-cell cultures were used to compare with co-cultures. For immortalised cell lines, cultures were performed in either mono-cell culture of BEAS-2B or a co-culture of BEAS-2B and A549. The mono-cell culture was performed with approximately 1 × 10^4^ BEAS-2B on the apical side of laminin (2.5 µg/mL) (synthesised by Prof JH Jang, Inha University, South Korea) coated transwell inserts (Corning) for a total of 11 days. Co-cultures were performed with either 1 × 10^4^ BEAS-2B on the apical side and 1 × 10^4^ A549 on the basal side or 1 × 10^4^ A549 on the apical side and 1 × 10^4^ BEAS-2B on the basal side. All cultures were maintained submerged in DMEM (Sigma-Aldrich, Australia) for four days, then switched to air–liquid interface (ALI) condition for seven days before harvesting (DMEM medium was used in the basolateral compartment, no medium was used in the apical compartment). Cultures were maintained either in static condition or dynamic condition using microfluidic CNBio’s PhysioMimix™ 3D system (clockwise direction, flow rate: 0.5 µL/s).

Similar to the immortalised cell cultures, primary human cell cultures were performed in either mono-cell culture of NHBE or co-culture of NHBE and NHLF. Mono-cell culture was performed with approximately 1 × 10^4^ NHBE on apical side, while co-culture was performed with approximately 1 × 10^4^ NHBE on apical side and 1 × 10^4^ NHLF on basal side. All the cultures were performed on the 0.4 µm transwell inserts (Corning) coated with laminin (2.5 µg/mL). For the diseased model, NHBE were replaced with disease human bronchial epithelial cells (DHBE). NHLF cells were maintained in FGM-2 medium (Lonza, USA) while either NHBE or DHBE cells were maintained in BEGM medium (Lonza, USA). All the cultures were maintained in medium for seven days, then switched to ALI condition using PneumaCult™-ALI medium (STEMCELL Technologies™, Australia) for three weeks. Cultures were maintained either in static condition or dynamic condition using microfluidic CNBio’s PhysioMimix™ 3D system (clockwise direction, flow rate: 0.5 µL/s).

#### Modification of the substrate with extracellular matrix (ECM) proteins

Extracellular matrix (ECM) plays an important role in cellular activity such as cell proliferation, differentiation, and migration [20]. Given that ECM influences the cellular activity and responses, it is necessary to examine the effect of different ECM in the development of the model. Therefore, two common ECM components were used to functionalise the substrates: (i) Collagen type I solution from rat tail (3 mg/mL) (Sigma-Aldrich, Australia), and (ii) fibronectin (2.5 µg/mL) (synthesised by Prof J.H Jang, Inha University, South Korea). To immobilise the collagen and fibronectin, the surfaces of the transwell membranes were activated using plasma cleaner for 5 min (PDC-002-HP, Harrick Plasma), and then incubated with either the solution of collage or fibronectin for 30 min at room temperature. After 30 min, the substrate solution was removed, and the membranes were primed with the culture medium for 1 h. Prior to seeding cells, the transwell membranes were washed with RNase-free PBS twice.

#### Validation of models using control/commercial SmallAir™-HF and MucilAir.™-HF models

For the validation purpose, all control/commercial co-culture models that mimic human airway epithelium (MucilAir™-HF) and small airway (SmallAir™-HF) were purchased from Epithelix Sárl, Geneva, Switzerland. MucilAir™-HF models were derived from freshly isolated primary human airway epithelial cells and fibroblasts from COPD donors. SmallAir™-HF models were reconstituted using isolated small airway human cells and fibroblasts from healthy donors. All the models were cultured in CNBio’s PhysioMimix™ at the ALI for three weeks using either MucilAir™ medium for MucilAir™-HF models or SmallAir™ medium for SmallAir™-HF models. Quality control was performed by the manufacturer shortly before shipment. After receipt, cell models were maintained for a total of four weeks (three weeks in ALI condition) before harvesting, with the medium replaced every two days.

### Quality control (QC) criteria for models

Since the models emulate human pathophysiology, we established seven quality control criteria for the models (Table 1). These criteria provide confidence that our models can be used as the standard platform for drug screening and disease modelling.

**Table 1:** Summary of quality control tests, purposes of the test and criteria for healthy models

*Quality control tests*	*Purposes of the test*	*Criteria for healthy lungs*
*Electrical resistance measurement (TEER measurement and impedance measurement)*	TEER measurement: To quantify the integrity of cellular barriers during the development stages Impedance measurement: To examine the growth and differentiation of the models as well as the integrity of cellular barriers of the models	TEER value falls into the range of 700–1200 Ω·cm^2^ [21] During the first stage of the growth of the models, the TEER value increases as the multilayers of cells are established in the models. During the second stage, the TEER value of the models decreases, and the capacitance of the models increases as the cells differentiate
*Light microscopy*	To observe and monitor the growth of cells during the cultures	High cellular confluency after a week of culture
*Permeability assay*	To assess the formation and function of cellular barriers	Decreased permeability of fluorescent dyes into the basal compartment
*Histology and immunofluorescence staining (IF) in cross-section view*	To visualise the cell layers in the cross-section view	Represent the key features of lungs such as ciliated cells, goblet cells, etc
*Immunofluorescence staining (IF)*	To detect and localise different antigens that are released from different types of cells and tissues in the models	Continuous and discrete tight junctions
*Scanning electron microscopy (SEM)*	To visualise the topological morphology and structure of the cells in the models	Fully ciliated cells and presence of goblet cells
*Cytokine profiling*	To quantify the expression level of released cytokines in the models	Different expression levels of cytokines in healthy and diseased models

#### Assessment of the integrity of the cellular barrier

##### TEER measurement

Assessment of barrier integrity is an important test to predict drug permeability, drug transport and drug interactions in humans [22]. To examine the integrity of cell layers and the development of tight junctions of all culture models, transepithelial electrical resistance measurement (TEER measurement) was performed every two days using an EVOM Volt/Ohm meter (World Precision Instruments WPI, Sarasota, USA). Both the apical and basolateral compartments were filled with fresh media to ensure sufficient medium for the measurement (250 μL of medium for the apical compartment and 750 μL of medium for the basolateral compartment). For ALI models, medium was then removed from apical compartment of the transwell inserts immediately after TEER measurement. The TEER values of models were corrected by deducting the background TEER values measured in the inserts with only the medium in both chambers (without cells). These values were then multiplied with membrane surface area (0.33 cm^2^) to achieve the unit area resistance (Ω·cm^2^).

##### Impedance spectroscopy

To examine the electrical resistance and capacitance of cell layers, impedance measurement was performed every two days using a high-throughput automated monitoring system – Locsense (Locsense, Netherland). Both the apical and basolateral compartments were filled with fresh media (250 μL of medium for apical compartment and 750 μL of medium for basolateral compartment). The impedance of cell layers was measured in a broad range of frequencies (from 10 to 100,000 Hz). The data were then exported in Microsoft Excel and analysed using GraphPad Prism.

#### Assessment of the cell growth during the culture

To assess the morphological features of the models during the culture such as confluency and integrity of cell layers, cell morphology, all the models were visualised every two days using a bright field phase-contrast inverted microscope Olympus CKX53 (Olympus Optical Co. Ltd., Tokyo, Japan).

#### Assessment of permeability of cellular barrier in the models

Permeability is one of the important assays to assess the function of lung models in numerous previous studies [23, 24]. Briefly, 1 mg/mL of FITC-Dextran (70 kDa) in cell culture medium (phenol red-free DMEM, FluoroBrite™ DMEM, ThermoFisher) was prepared. Cell culture medium in the apical and basolateral compartments of transwell inserts were removed. The transwell inserts were then transferred to a new 24-well plate. In that 24-well plate, 800 μL of FluoroBrite™ DMEM was added in the basolateral compartment and 250 μL of FITC-Dextran 1 mg/mL solution was added in the apical compartment of inserts. The plate was placed in a 37 °C incubator and protected from light, for 30–40 min. After incubation, the medium in the basolateral compartment was collected for analysis. Inserts without cells were used as a positive control, while cell culture media without FITC-Dextran was used as negative control. Media were then transferred from the basolateral compartment to a black 96-well microplate. Fluorescence from the medium was measured using a Victor X plate reader (PerkinElmer). The excitation and emission wavelength for the measurement of FITC-Dextran were 485 and 535 nm, respectively.

#### Assessment of structure of multilayers of the models and tight junctions on the apical side of the models

To assess the composition and structure of the models, classical hematoxylin/eosin (H&E) histological studies were performed on each model. When the models were ready to harvest (after 25 days of culture), the models were washed with PBS and fixed with 10% formalin for 24 h. Then, the models were washed with PBS and stored in PBS before processing. Only models derived from primary cell cultures were processed with H&E staining.

The specimen preparation for histology on the models were performed at the Anzac Research Institute, Australia. Briefly, the membranes of the models were detached from the inserts, embedded in paraffin, sectioned, and processed by standard H&E staining to examine the structure and visualise the composition of layers of the models [25].

Immunofluorescence (IF) staining was performed for cross-sectioned lung models. After sectioning the models, the permeabilization and antigen retrieval of the models were performed using citrate buffer, pH 6.0. The models were then incubated with a blocking solution containing 1% (w/v) BSA (ThermoFisher) and 10% (v/v) goat serum (ThermoFisher) to avoid nonspecific binding of the antibodies. The primary antibodies (including antibodies already conjugated with a fluorophore) were incubated overnight at 4 °C, and the appropriate secondary antibodies were incubated the next day for 60 min at room temperature (Table S1). Models were stained with DAPI to visualise the nuclei of the cells. Subsequently, the models were mounted onto the glass slide and fluorescence labelling was visualised using a Zeiss fluorescent microscope (Zeiss Axioscope 5).

IF staining was also performed on the apical side of the models to assess the formation of tight junctions. Briefly, the models were washed with PBS and fixed with 4% paraformaldehyde for 15 min. The models were then stained with primary antibodies monoclonal ZO-1 (clone: ZO1-1A12, catalogue number: 339100, ThermoFisher) and secondary antibodies Goat Anti-mouse IgG (H + L), F(ab’)2 Fragment conjugated to CF633 (catalogue number: 20130–1, Assay Matrix Pty Ltd). DAPI was added to stain the cell nucleus. Subsequently, the membranes of the models were carefully detached from the inserts. The membranes were mounted with Fluoromount™ Aqueous mounting medium (cat: F4680, Sigma Aldrich) on a glass slide and visualised using Nikon A1 or Nikon C2 confocal microscope.

#### Assessment of cell morphology on the apical side of the models

The structure and morphology of cells on the surface of the models are important to define the health status of the models. Scanning electron microscopy (SEM) was used to visualise the morphology of cells on the apical side of membrane inserts. Briefly, the models were washed twice with PBS and fixed with 2.5% glutaraldehyde in PBS (cat: G5882, Sigma Aldrich) for 2 h. Models were washed with PBS for three times, each time for 5 min. Next, the membranes of the models were cut from the insert, dehydrated in graded ethanol solutions (from 30 to 100% ethanol) and dried using a critical point dryer (CPD300, Leica Microsystems). Models were then coated with gold at 10 nm coating thickness. Subsequently, the samples were kept in a desiccator filled up with silica gel prior to SEM imaging. SEM imaging was performed using Zeiss Sigma HD FEG SEM. Secondary electrons were acquired using the detectors of the SE2 mode for all models with an accelerating voltage of 5.0 kV and a working distance (W.D) = 8.0.

#### Cytokine profiling

To characterise the cytokine secretion profile of the lung models, culture supernatant from the basolateral compartment was collected at the end of culture and stored at -80 °C until processing. Culture supernatants were thawed, and the levels of cytokines measured using the Bio-Plex Pro™ Human Cytokine 27-plex (#M500KCAF0Y, Bio-Rad) run according to the manufacturer’s recommendations. Data was obtained using a MAGPIX Bead Assay Reader (Luminex) using xPONENT software (Build 4.2.1324.0, Luminex).

#### Statistical analyses

Data was analysed and presented as mean ± standard deviation. For developed models, a minimum of three separate replicates were used per experiment (*n* = 3). Statistical analysis was performed using one-way ANOVA followed by Dunn’s multiple comparison’s test for pair-wise comparisons. A *P*-value < 0.05 was considered to be statistically significant. For cytokine profiling, a one-tailed T-test was used (*n* = 4).

## Results

### Incorporation of co-culture of two immortalised lung cell lines (A549 and BEAS-2B) and dynamic flow improved the integrity of the cellular barrier in the lung models

First, we attempted to optimise the culture condition (dynamic *vs* static) using two immortalised lung cell lines (A549 and BEAS-2B). The dynamic condition was created using the microfluidic 3D system – CNBio’s PhysioMimix™. This system allows the continuous perfusion of media with the flow rate at 0.5 µL/s in the basolateral compartments of the inserts. For static condition, the cell cultures were maintained in normal 24 well plates without any media perfusion.

The complexity was incorporated to recapitulate the cell–cell interaction in human lungs. To increase the complexity, BEAS-2B and A549 cells were selected for the co-culture model. To identify which cells should be seeded on the apical and basal side, two co-culture models were tested: BEAS2B-A549 (BEAS-2B cells were seeded on the apical side, A549 cells were seeded on the basal side) and A549-BEAS2B (A549 cells were seeded on the apical side, BEAS-2B cells were seeded on the basal side).

**Fig. 2:**
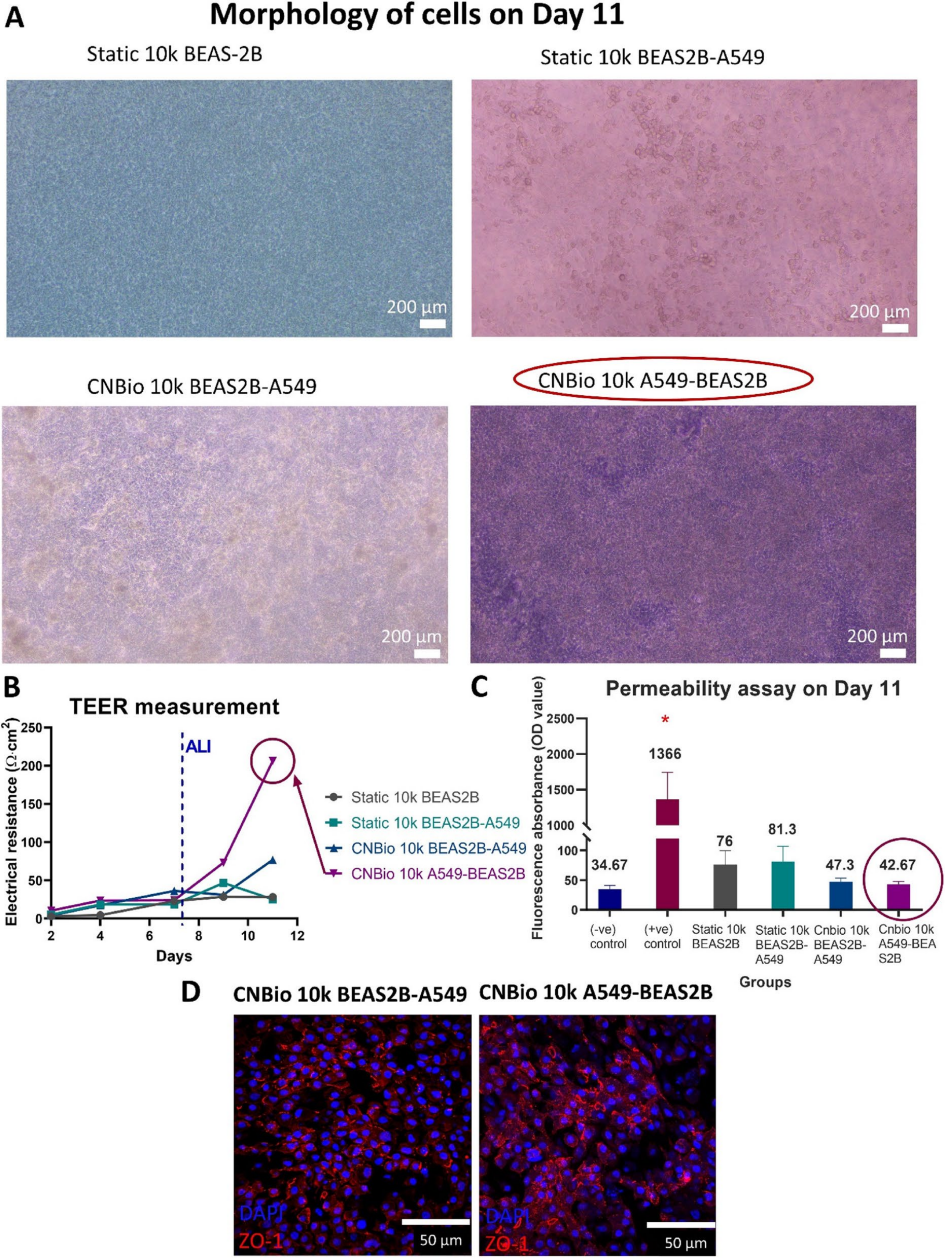
Morphological appearance, electrical resistance, and FITC-Dextran permeability of the optimised models (static 10,000 BEAS-2B cells, static 10,000 BEAS2B-A549, CNBio BEAS2B-A549 and CNBio 10,000 A549-BEAS2B). **A** The morphology of cells at the end of the culture for all the models on Day 11. Scale bar represents 200 µm; **B** The electrical resistance of all the models over a period of 11 days where ALI was performed on Day 4; **C** The permeability of FITC-Dextran into the basolateral compartment of all the models. Negative (-ve) control was the medium only without FITC-Dextran. Positive (+ ve) control was empty insert without cells followed the same protocol as the inserts with cells. Mean ± SD are plotted. Statistical significance was tested using ordinary one-way ANOVA, followed by Dunn’s multiple comparison’s test for pair-wise comparisons, *n* = 3. **D** Representative IF staining of CNBio 10,000 BEAS2B-A549 and CNBio 10,000 A549-BEAS2B showing disrupted tight junctional proteins ZO-1 (red) and DAPI (blue); scale bar, 50 µm

Four models from simple to complex condition were tested. They were: (i) mono-cell culture of BEAS-2B in static condition (static 10,000 BEAS-2B); (ii) co-culture of BEAS-2B (apical side) and A549 (basal side) in static condition (static 10,000 BEAS2B-A549); (iii) co-culture of BEAS-2B (apical side) and A549 (basal side) in dynamic condition (CNBio 10,000 BEAS2B-A549); (iv) co-culture of A549 (apical side) and BEAS-2B (basal side) in dynamic condition (CNBio 10,000 A549-BEAS2B) (Fig. 2). The morphology of cells in these models was taken every two days to monitor the growth of cells. After 11 days, all four models formed confluent layer of cells (Fig. 2 A). Regarding the integrity of the cellular barrier, CNBio 10,000 A549-BEAS2B had the highest electrical resistance value among four models (~ 200 Ω·cm^2^), followed by CNBio 10,000 BEAS2B-A549 (~ 100 Ω·cm^2^), and static 10,000 BEAS-2B and static 10,000 BEAS2B-A549 (~ 50 Ω·cm^2^) (Fig. 2B). Overall, the static culture had a lower electrical resistance value when compared with the dynamic models (CNBio models), which indicated that the dynamic flow improved the cellular barrier integrity.

The importance of selecting which cell line should be seeded in the basal or apical side of the insert was shown in the comparison between dynamic models CNBio BEAS2B-A549 and CNBio A549-BEAS2B. The electrical resistance value of CNBio A549-BEAS2B was higher (around 300 Ω·cm^2^) than CNBio BEAS2B-A549 (~ 100 Ω·cm^2^), which was consistent with the lowest permeability of FITC-dextran in CNBio BEAS2B-A549 (Fig. 2C). Therefore, the location where the cells should be seeded is important in the development of lung models. There was no statistical significance between all the models and negative control, which confirms their low permeability and proper barrier function.

To investigate whether CNBio A549-BEAS2B can recapitulate the tight junctions in human lungs, the ZO-1 – an intracellular protein of the tight junction complex – was used as a marker. CNBio BEAS2B-A549 was used as a secondary model to compare with CNBio A549-BEAS2B. The structure of tight junctions for both CNBio A549-BEAS2B and CNBio BEAS2B-A549 models was non-uniform and fragmented (indicated in red colour in Fig. 2D), which indicated that these models cannot form the appropriate tight junctions.

### Replacing the immortalised lung cell lines (BEAS-2B, A549) with the primary lung cell lines (NHBE, NHLF and COPD-NHBE) improved the cellular barrier and developed important structures such as the tight junctions, ciliated cells, and goblet cells

Since the most promising CNBio A549-BEAS2B model could not form proper tight junctions, the next step was to optimise the cell lines. To optimise the cell lines, human bronchial epithelial cells (NHBE) and human lung fibroblasts (NHLF) were used in this study. NHBE and NHLF were used to mimic the human lung environment and reconstitute the human respiratory mucosa [26]. Fibroblasts can support the epithelial cell function by promoting proliferation and differentiation, modulating mucin secretion, and triggering a correct spatial distribution [26]. Hence, fibroblasts can contribute to an appropriate assembling of the bronchial epithelium and aid to maintain the mucociliary phenotype for a long duration.

**Fig. 3:**
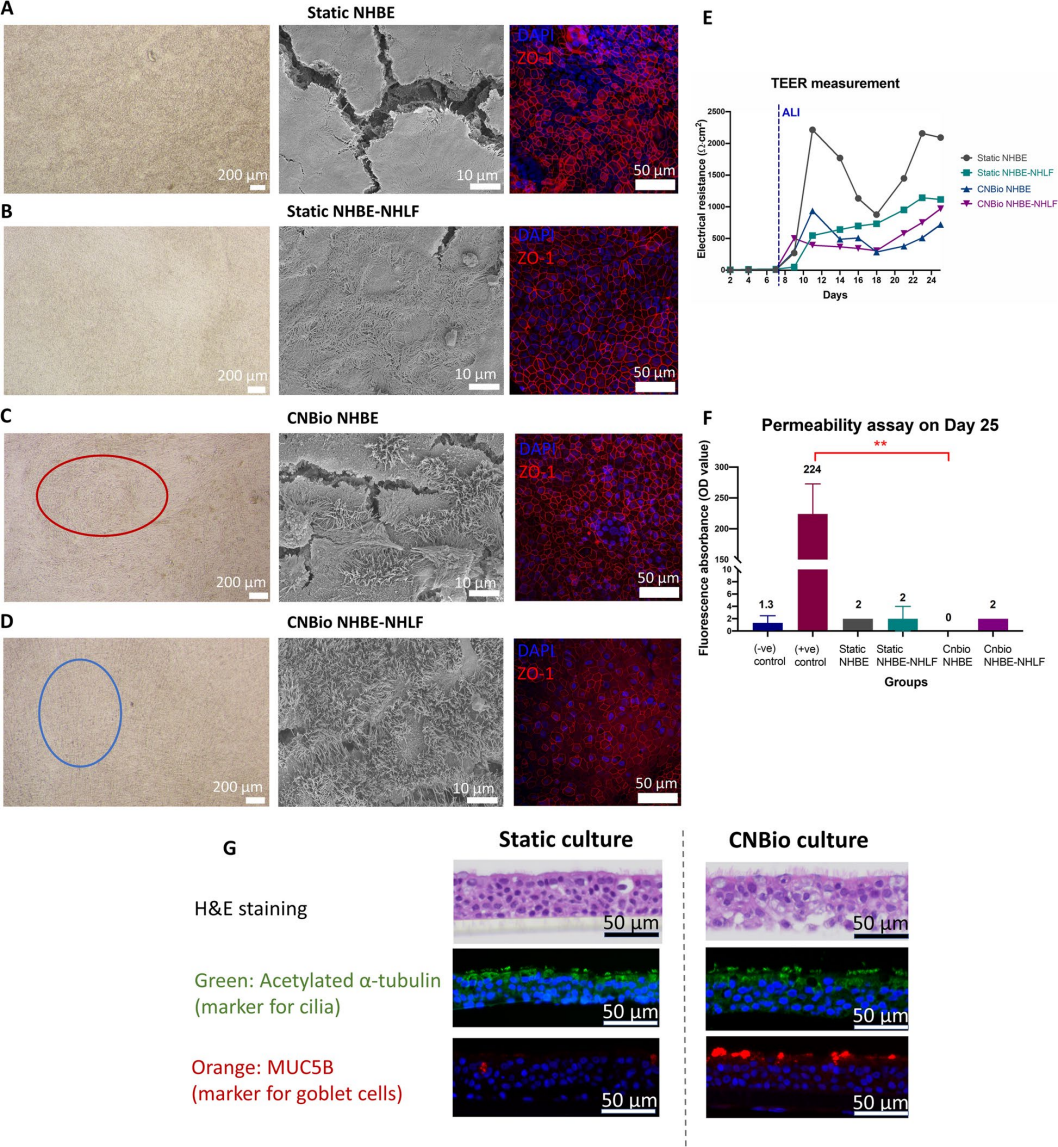
Comparison of morphological appearance, barrier integrity, permeability and structure of developed models using human primary cell lines. Representative morphological appearance, SEM images and IF staining of developed models; **A** static mono-cell culture (NHBE), **B** static co-culture (NHBE-NHLF), **C** dynamic mono-cell culture (CNBio NHBE), **D** dynamic co-culture (CNBio NHBE-NHLF) on the Day 25. The morphology of cells at the end of culture (Day 25 of culture). Scale bar represents 200 µm. For SEM, images showed the morphology of cells on the apical surface of the model; scale bar, 10 µm. For IF staining, ZO-1 (red) and DAPI (blue) staining were used to demonstrate the continuous tight junctional connections; scale bar, 50 µm. **E** The electrical resistance of the models over a period of 25 days of culture; ALI condition was performed on Day 7 for all models; **F** The permeability of FITC-Dextran into the basolateral compartment of all the models. Negative (-ve) control was the medium only without FITC-Dextran. Positive (+ ve) control was the empty insert without cells followed the same protocol as the inserts with cells. Error bars denote standard deviation. Statistical significance was shown between positive (+ ve) control and CNBio NHBE; **: *P* < 0.01. **G** Representative histological cross-sections (H&E staining) and IF staining display essential epithelial features, including acetylated α-tubulin (green, marker for ciliated cells) and MUC5B (red, marker for goblet cells) for the static culture and dynamic culture. Objective 40x, scale bar represents 50 µm

For optimisation purposes, four models were developed: static mono-cell culture of NHBE (static NHBE), static co-culture of NHBE and NHLF (static NHBE-NHLF), dynamic mono-cell culture of NHBE (CNBio NHBE) and dynamic co-culture of NHBE and NHLF (CNBio NHBE-NHLF). The morphology of these models after 25 days is presented in Fig. 3. All models showed confluent layers of cells, however, the phenotype of cells in dynamic models (both mono-cell culture and co-culture models) was more heterogenous (as shown in red and blue circles). This could be due to the flow in perfusion system, which might impact on the growth of cells.

All four models (static NHBE, static NHBE-NHLF, CNBio NHBE, CNBio NHBE-NHLF) exhibited highly organised tight junctions with ZO-1 detected in a discrete and continuous localisation around the periphery of the cells (Fig. 3). This result suggested that using primary cell lines can improve the tight junctions between the cells in comparison to immortalised cell lines. Moreover, SEM data showed that both mono-cell culture and co-culture models established under dynamic conditions retained the ‘hairy’ structure of ciliated cells on the surface. Whereas, in the static condition (for both mono-cell culture and co-culture), there was a lack of protruding cilia from the surface of the models (Fig. 3). This result suggested that both microcirculation (dynamic media flow) and the use of two key cell types present in lung tissues (NHBE and NHLF) are essential to promote the formation of a lung-like structure that emulate some of the physiological lung functions, such as barrier protection and mucociliary clearance.

The electrical resistance of all four models (in the range of 500 to 2000 Ω·cm^2^) (Fig. 3E) was higher than CNBio A549-BEAS2B model (~ 300 Ω·cm^2^) (Fig. 2B). In addition, low permeability of FITC-dextran was shown in all four models (Fig. 3F), which indicated the formation of a proper cellular barrier.

In addition, the histological cross-section images were consistent with SEM images showing more cilia in the dynamic culture than in the static culture (Fig. 3G). The histological cross-section of models showed cells form multilayers, suggesting that all the models were composed of fully differentiated airway epithelial cells (Fig. 3G). Especially, the expression of markers for cilia (acetylated α-tubulin) and goblet cells (MUC5B) was higher for dynamic cultures compared to static cultures, which suggested that the dynamic condition supported the differentiation and development of the models.

Taken together, these data suggest that the dynamic co-culture of primary lung cell lines (NHBE and NHLF) would be the most appropriate model to mimic the living healthy human lungs.

### CNBio NHBE-NHLF displayed similar composition and structure as commercial healthy lung model

**Fig. 4:**
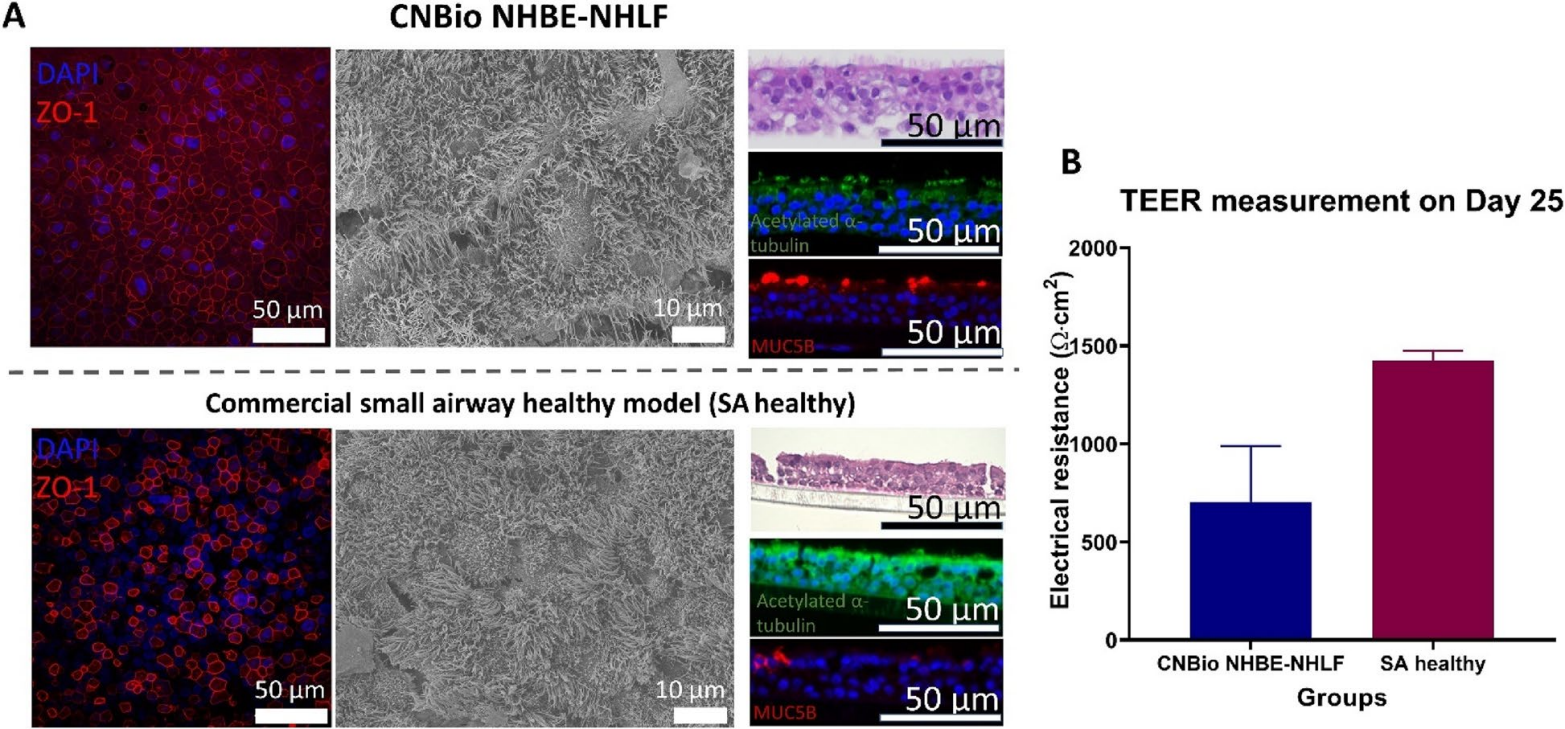
Comparison between CNBio NHBE-NHLF and commercial SmallAir™-HF derived from healthy donors, regarding (**A**) IF staining (ZO-1: red, DAPI: blue); Scale bar: 50 µm, SEM images, and histological cross-sections (H&E staining) and IF staining displaying essential epithelial features, including acetylated α-tubulin (green) – marker for ciliated cells and MUC5B (red) – marker for goblet cells for the static culture and dynamic culture; Objective 40x, scale bar represents 50 µm. **B** The electrical resistance value at the end of the culture (Day 25). Error bars denote standard deviation, *n* = 6 for CNBio NHBE-NHLF and *n* = 2 for SmallAir™-HF derived from healthy donors

To validate the structure and functionality of CNBio NHBE-NHLF, the commercial small airway healthy model SmallAir™-HF (SmallAir™-HF healthy), which comprised human airway epithelial cells and fibroblasts from healthy donors, was used. Both models expressed discrete tight junction ZO-1. However, CNBio NHBE-NHLF expressed more continuous ZO-1 expression than SmallAir™-HF healthy (Fig. 4A). Fully ciliated cells were shown in SEM images and multilayers of cells were shown in cross section histological images in both models (Fig. 4A). Moreover, CNBio NHBE-NHLF developed a defined cilia structure (acetylated α-tubulin – green) and goblet cells (MUC5B – red) when compared to SmallAir™-HF healthy. This result suggested that these models can mimic the important features of the human lungs. Importantly, the electrical resistance of these models was high (around 700–1400 Ω·cm^2^), in which the control SmallAir™-HF healthy had a higher value (1400 Ω·cm^2^) than developed model CNBio NHBE-NHLF (700 Ω·cm^2^) (Fig. 4B). These results confirmed that the structure of our developed model closely resembled the tissue architecture of in vivo airway epithelium.

### Different coating substrates and different types of transwell inserts affected the cellular barrier integrity, the growth and differentiation of CNBio NHBE-NHLF

**Fig. 5:**
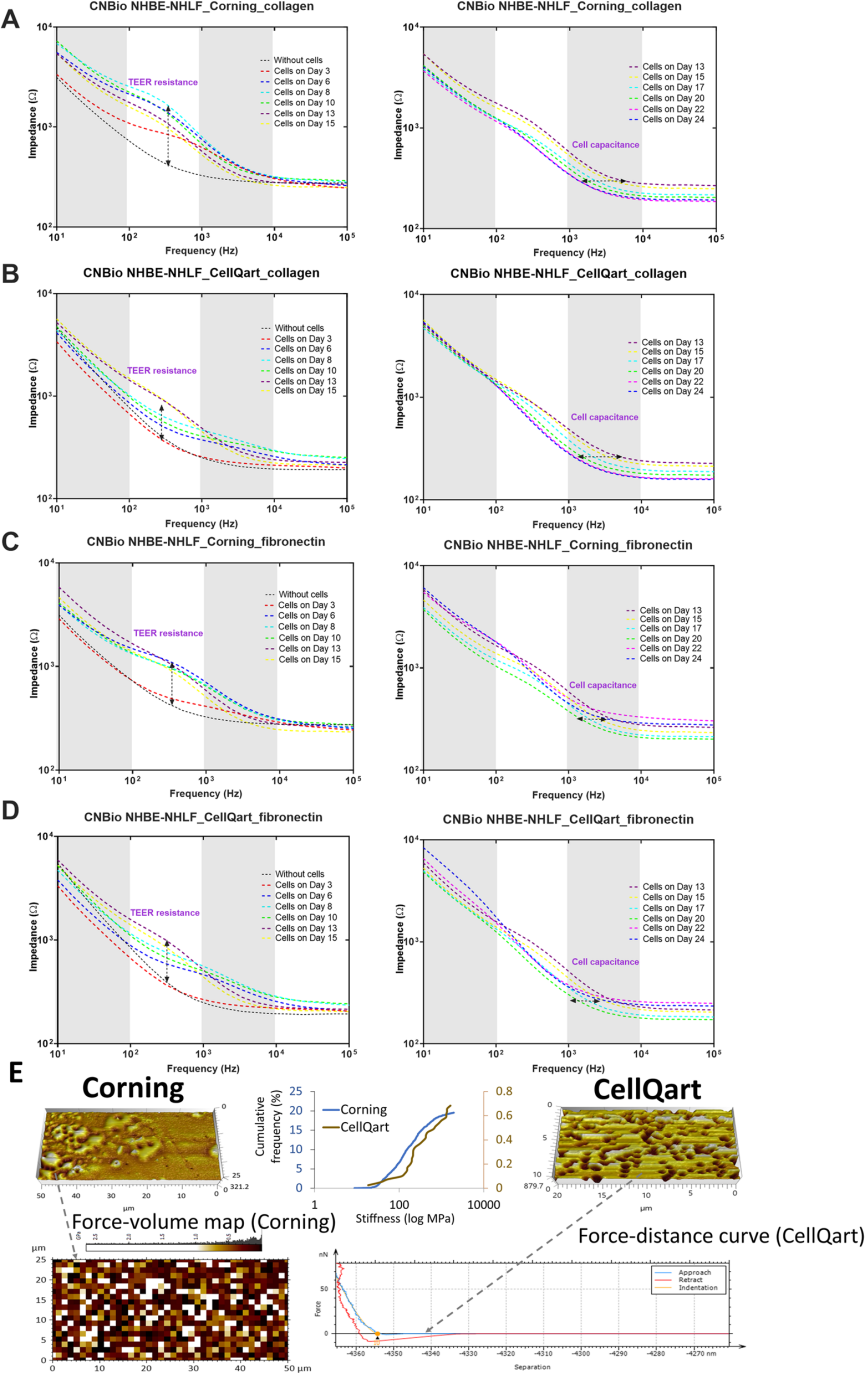
The impedance measurement of CNBio NHBE-NHLF models was performed every 2–3 days during 24 days. The left panels indicated the TEER value and the right panels indicated cell capacitance. The TEER value (from Day 3 to Day 15) and cell capacitance (from Day 13 to Day 24) were presented for (**A**) CNBio NHBE-NHLF coated with collagen on the Corning insert (**B**) CNBio NHBE-NHLF coated with collagen on the CellQart insert (**C**) CNBio NHBE-NHLF coated with fibronectin on the Corning insert (**D**) CNBio NHBE-NHLF coated with collagen on the Corning insert. *n* = 3. **E** 3D topographical AFM images and linear regression analysis of the stiffness of the Corning and CellQart inserts. The stiffness analyses of the Corning and CellQart inserts were generated from the force-volume map of the Corning insert (800 points) and 40 individual force-distance curves of the CellQart insert

Two common coating substrates – collagen and fibronectin – and two transwell membranes with different pore-density – Corning and CellQart – were used to optimise the most appropriate healthy lung model CNBio NHBE-NHLF. To monitor the cellular barrier integrity of these models, we used an impedance measurement system, which allows real-time readouts of the cellular barrier function of the models. Unlike the TEER measurement which occurs at one single frequency, impedance measurement allows the measurement of impedance across a wide range of frequencies. This impedance measurement provides not only the barrier function of cell layers (TEER) but also the process of cell growth and cell differentiation in the model (cell capacitance). The TEER value of the model is calculated based on the differences in height between the curve in the models (with cells) and control (without cells) (as shown in the black arrow in the left panels in Fig. 5). The increase in TEER value of the model shifts the plateau of the curve upwards. The capacitance of the model is indicated by the width of the curve of the models (as shown in the black arrow in the right panels in Fig. 5). The increase in capacitance of the model narrows the plateau of the curve.

The impedance measurements for each model were carried out for 24 days. In the first 15 days of the culture TEER was a dominating signal and its gradual increase was associated with the formation of both tight junctions and multilayer structure (Day 3 – 15, Fig. 5 left panels). Since day 13 we observed an increase and dominance of capacitance signal, which was related to the differentiation of the cells (Day 13 –24, Fig. 5 right panels). Regardless of the membrane coating, collagen or fibronectin, TEER values for the models established on Corning inserts were higher than for CellQart inserts (Fig. 5 left panels). On Day 3, there was a substantial difference between TEER values recorded for the models established on Corning inserts with collagen- and fibronectin-coated membranes (140 Ω·cm^2^ and 20 Ω·cm^2^ respectively). Substantial differences in TEER values suggested that collagen promoted adhesion of cells and rapid formation of cell monolayers. Between Day 3 and 8 TEER of both models gradually increased and reached 442 Ω·cm^2^ (threefold increase) and 187 Ω·cm^2^ (ninefold increase) respectively for collagen- and fibronectin-coated membranes; this result suggested that the fibronectin was more effective in increasing the growth rate of cells than collagen. In contrast, there were no statistically significant differences in the TEER between the models formed on CellQart inserts with both types of coating in the same time frame. For example, on Day 8, the TEER value of the models established on CellQart inserts coated with collagen was 80 Ω·cm^2^ and fibronectin 121 Ω·cm^2^. Relatively constant TEER of both CellQart models could be associated with the uniform structure of the CellQart membranes and potentially more uniform and functionally immobilised coatings of both collagen and fibronectin.

On Day 10, TEER of all models dropped by 25%, which could be due to the change in culture condition from the liquid phase to the air–liquid interface (ALI) and could be related to the cell differentiation. From Day 10 to Day 15, TEER values for the models formed on Corning inserts continued to decrease, which suggested that the cells underwent unspecific differentiation, which interfered with the TEER. Specifically, from Day 10 to Day 15, TEER for the models grown on Corning inserts dropped around 1.5-fold and reached similar value on Day 15 (198 Ω·cm^2^ and 172 Ω·cm^2^ for collagen- and fibronectin-coated membranes respectively). This result indicated that the type of coating did not affect the cell growth at the latter time point (in ALI), potentially due to the fact that the monolayers were already established.

TEER for the models grown on CellQart inserts coated with collagen and fibronectin increased steadily from Day 10 to 15. This result suggested that the cells continued to form physiological structures (cell layers). There were no significant differences of TEER recorded at Day 15 for the models coated with collagen (174 Ω·cm^2^) and fibronectin (153 Ω·cm^2^), meaning that the cells continued to differentiate into a uniform structure with physiological tight junctions.

In summary, a fluctuation of TEER observed in Corning models (a rapid increase for first eight days and a gradual decrease from Day 10 to Day 15) may be attributed to unspecific cell differentiation and disrupted tight junction structure. Meanwhile, a steady increase in TEER observed in CellQart models suggested that a uniform and robust tight junction structure was formed. In addition, a higher TEER for collagen-coated membranes, regardless of the type of inserts, suggested that collagen is effective in supporting cell adhesion and growth.

Regardless of the insert types, the models coated with collagen showed an increase in cell capacitance from Day 13 to Day 24 (Fig. 5A, B right panels). Whereas the models coated with fibronectin showed an increase in cell capacitance from Day 13 to Day 20, a decrease from Day 20 to Day 22 and an increase from Day 22 to Day 24 (Fig. 5C, D right panels). This result suggested that the collagen promoted better growth and differentiation of the models than fibronectin, which was consistent with the observation in TEER.

To assess topography of the membranes and to measure their mechanical properties, Atomic Force Microscopy (AFM) and nanoindentation was used. The topological image of the CellQart insert showed a uniform and highly porous structure, where porous of sizes ~ 0.5 μm was uniformly distributed (Fig. 5E). In contrast the topography of the Corning insert showed less porous and heterogenous structure. Number, size, and uniform distribution of pores in the CellQart insert guarantees continuous nutrients delivery to cells, as well as more physiological media and paracrine signal exchange between cells that are grown on both sides of the membrane. The structure of the Corning insert, on the other hand, characterises with lower number and heterogenous pores of small size, suggesting that there are limited media and nutrients exchange between two sides of the membrane. The nanoindentation results showed that the average Young’s modules of the CellQart and Corning inserts were 511 ± 482 kPa and 266 ± 315 kPa, respectively and statistical analysis showed no statistically significant differences in the stiffness between the CellQart and Corning inserts. Since the stiffness is one of the key parameters that modulates cell responses and differentiation [27], the biomechanical properties of the membranes was further modulated by functionalising their surfaces with ECM. Cumulatively, functionalised membranes provided biomechanical and biochemical cues to enable desired cell differentiation and growth, thus functionality of the models.

### Using diseased human bronchial/tracheal epithelial cells—COPD (DHBE) was effective in recapitulating the features observed in COPD patients

**Fig. 6:**
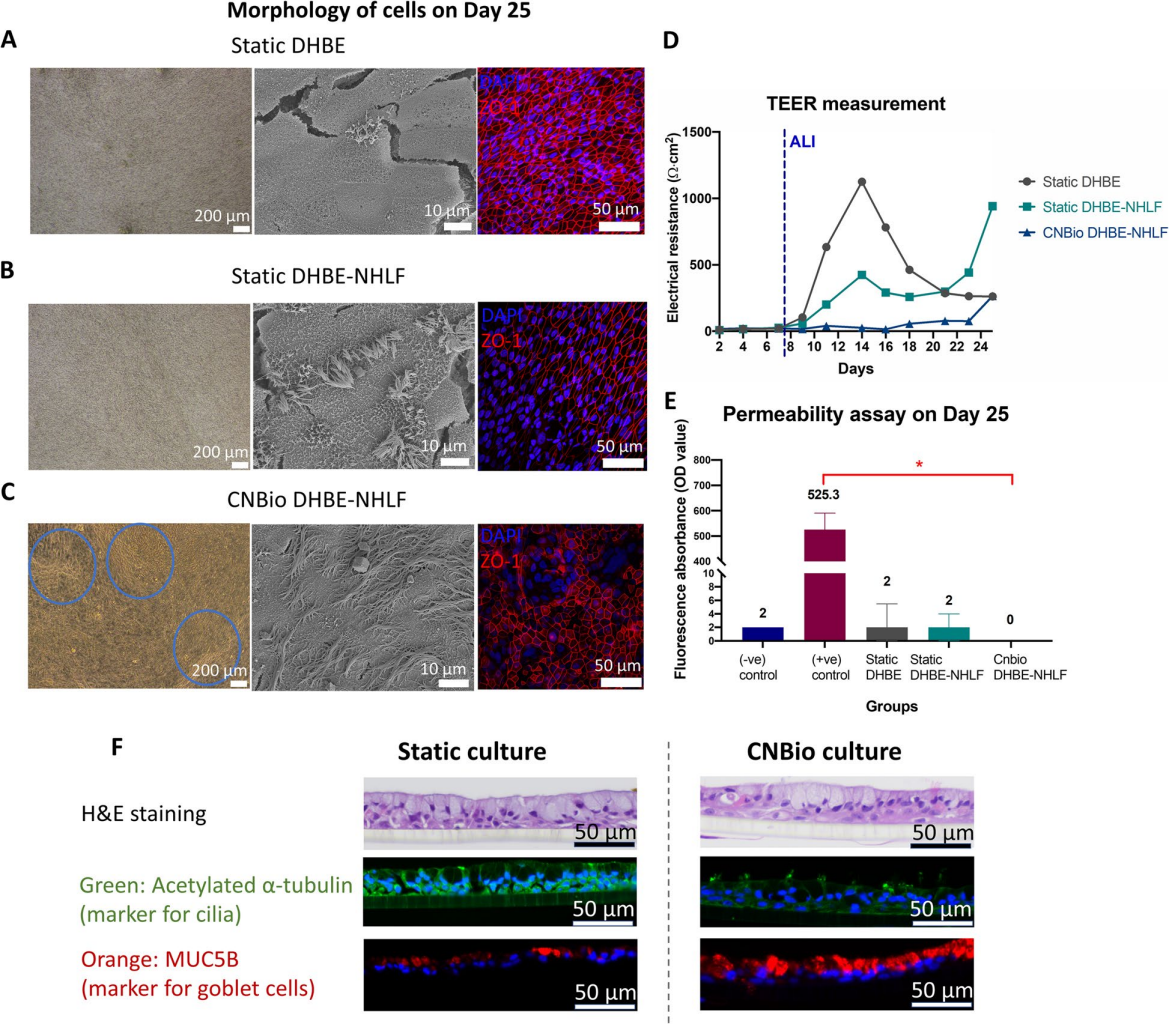
Morphological appearance, barrier integrity, permeability, and the structure of static mono-cell culture (DHBE), static co-culture (DHBE-NHLF) and dynamic co-culture (CNBio DHBE-NHLF) disease models. **A** The morphology of cells at the end of culture (on the Day 25 of culture). Scale bar represents 200 µm; and representative SEM images (scale bar, 10 µm) and IF staining show the tight junctions of disease models including static mono-cell culture (DHBE), static co-culture (DHBE-NHLF), and dynamic co-culture (CNBio DHBE-NHLF) on the Day 25 (scale bar, 50 µm). **B** The electrical resistance of disease models was measured over the course of 25 days of cell culture; ALI condition was performed on Day 7 for all disease models; **C** The permeability of FITC-Dextran into the basal side of all the models. Negative (-ve) control was cell culture media only, without FITC-Dextran. Positive (+ ve) control was empty insert without cells. Error bars indicate for standard deviation. Statistical significance was shown between (+ ve) control and CNBio DHBE-NHLF; *: *P* < 0.05. **D** Cross-section histological representative images and IF staining displaying essential epithelial features, including acetylated α-tubulin (green) – marker for ciliated cells and MUC5B (red) – a marker for goblet cells for the static and dynamic culture models. Objective 40x, scale bar represents 50 µm

To develop the disease models, the diseased primary lung cell line (DHBE) was used. Similar to the healthy models, three disease models were established and compared: static mono-cell culture of DHBE (static DHBE), static co-culture of DHBE and NHLF (static DHBE-NHLF), and dynamic co-culture of DHBE and NHLF (CNBio DHBE-NHLF). All disease models were developed using traditional Corning inserts. A confluent monolayer of cells after 25 days of culture was shown in all disease models (Fig. 6A). However, a more heterogenous cell structure was developed in the CNBio DHBE-NHLF model (as indicated in the blue circles). This again highlighted the impact of the flow on the growth and morphology of cells. The electrical resistance of static DHBE-NHLF was highest (941 Ω·cm^2^), followed by CNBio DHBE-NHLF and static DHBE (~ 250 Ω·cm^2^) (Fig. 6B). Low permeability of FITC-dextran was shown in all disease models (Fig. 6C), which suggested that a proper cellular barrier was formed in all disease models. There was significant difference between positive control and CNBio DHBE-NHLF (Fig. 6C).

After the assessment of cellular barrier and permeability, the expression of tight junction ZO-1 in static DHBE, static DHBE-NHLF and CNBio DHBE-NHLF was evaluated. The expression of tight junctions in the mono-cell model (static) was different from co-culture models (static and dynamic) (Fig. 6A). The static mono-cell culture DHBE expressed discrete and continuous tight junctions, whereas the co-culture static DHBE-NHLF and CNBio DHBE-NHLF had discontinuous and altered localisation of proteins. The differences between the models were also revealed in SEM images (Fig. 6A). SEM images of the static DHBE and DHBE-NHLF presented a small number of cilia, while the representative SEM image of the apical side of the CNBio DHBE-NHLF model showed more presence of cilia (Fig. 6A). Despite the differences in tight junctions and morphology of cells on the apical sides of the models, the number of cilia in disease models (Fig. 6A) was significantly reduced in comparison to healthy models (Fig. 4). Additionally, the thickness of the disease models was smaller than the healthy models, which confirmed the impact of disease on the differentiation and growth of the models. Notably, the model grown under dynamic conditions exhibited higher expression of goblet cells when compared to the model grown in static conditions (Fig. 6D). Since the high expression of goblet cells is one of the features in COPD patients, this result suggested that the cells cultured under dynamic condition can recapitulate the hallmarks of diseased human lungs.

**Fig. 7:**
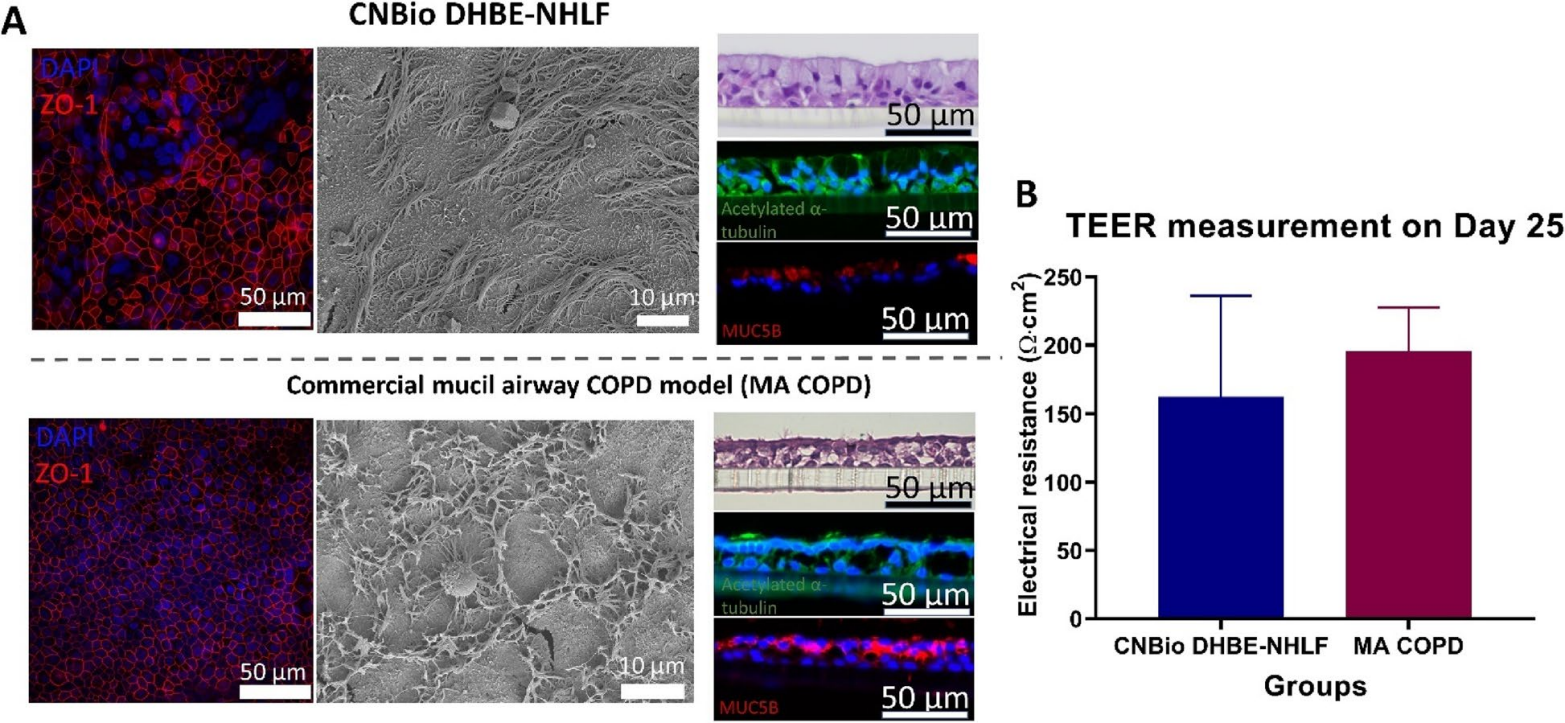
Comparison between developed disease lung model and commercial MucilAir™-HF derived from COPD donors. **A** IF staining (ZO-1: red, DAPI: blue); Scale bar: 50 µm, SEM images, and histological cross-sections (H&E staining) and IF staining displaying essential epithelial features, including acetylated α-tubulin (green) – marker for ciliated cells and MUC5B (red) – marker for goblet cells for the static culture and dynamic culture; Objective 40x, scale bar: 50 µm. **B** the electrical resistance value at the end of the culture (Day 25). Error bars denote standard deviation, *n* = 3 for CNBio NHBE-NHLF and *n* = 2 for MucilAir™-HF derived from COPD donors

The MucilAir™-HF derived from COPD patients (MucilAir™-HF COPD) was used to compare against the developed disease model (CNBio DHBE-NHLF). Both models expressed a reduced numbers of cilia in comparison to healthy models as shown in SEM images and IF staining of acetylated α-tubulin in cross-section (Fig. 7A). However, there were differences in the expression of tight junctions and the composition presented in cross-section histology between the developed disease model (CNBio DHBE-NHLF) and control MucilAir™-HF COPD model (Fig. 7A). While CNBio DHBE-NHLF expressed discontinuous tight junctions, control MucilAir™-HF COPD had discrete, continuous tight junctions. The cross-section histological images of these models were also different. While CNBio DHBE-NHLF expressed overproduction and hypertrophy (increase in size) of goblet cells, MucilAir™-HF COPD did not exhibit the hypertrophy of goblet cells. The hypertrophy of goblet cells was also observed in another static culture disease model (Fig. 6D). In addition, the electrical resistance value of the MucilAir™-HF COPD model (around 200 Ω·cm^2^) was higher than the CNBio DHBE-NHLF (around 160 Ω·cm^2^) at the end of culture (Fig. 7B). The differences between CNBio DHBE-NHLF and MucilAir™-HF COPD models are likely to be associated with three key factors: (1) the design of the model, (2) the culture condition and (3) the cellular composition.

### The level of pro-inflammatory cytokine IP-10 and IL-6 is significantly higher in the developed disease model than healthy model

**Fig. 8:**
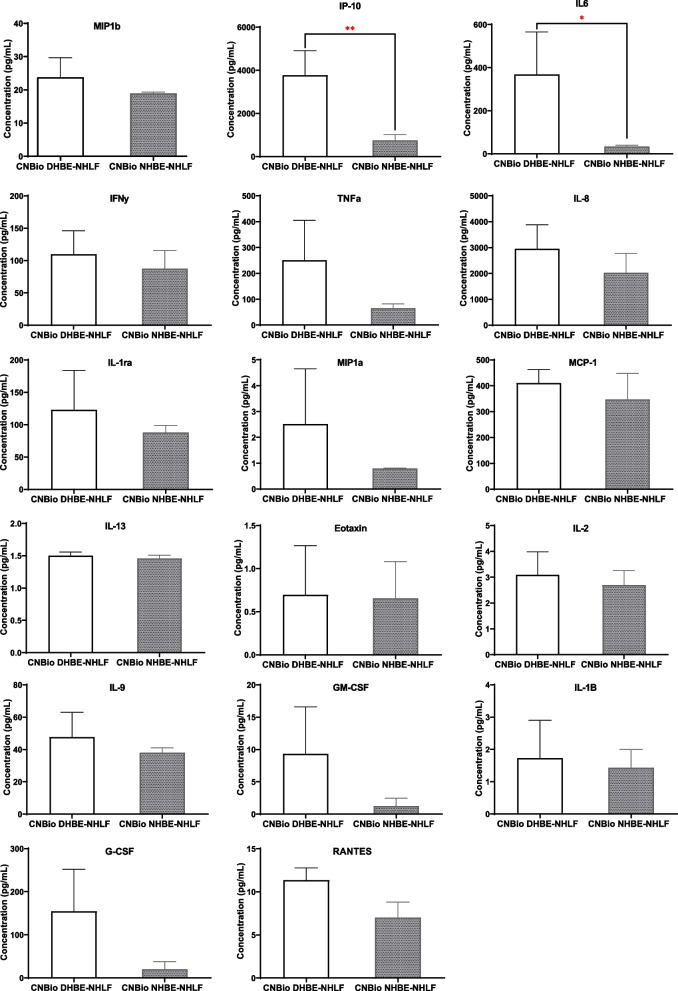
The production of cytokines in disease (CNBio DHBE-NHLF) and healthy (CNBio NHBE-NHLF) models. Statistical significance was assessed using a one-tailed T-test, *n* = 4. *: *P* < 0.05, **: *P* < 0.01

For a better understanding of the inflammation process in the lung models, profiling of the cytokine produced by the healthy and disease models was performed. Chemoattractant cytokines (chemokines), pro- and anti-inflammatory cytokines can affect the host response to inflammation by mediating the activation of leukocytes. Therefore, the increase or decrease levels of these cytokines can be used as an early marker for lung inflammation in COPD [28]. Hence, the production of 27 cytokines was measured using Luminex bead-based assay for developed disease (CNBio DHBE-NHLF) and healthy (CNBio NHBE-NHLF) models (Fig. 8). Overall, the expression level of all cytokines in disease CNBio DHBE-NHLF was higher than healthy CNBio NHBE-NHLF. Importantly, the level of pro-inflammatory cytokine IP-10 and IL-6 increased significantly in disease CNBio DHBE-NHLF in comparison to healthy CNBio NHBE-NHLF (Fig. 8). This result suggested that the pro-inflammatory cytokine IP-10 and IL-6 could be early markers for COPD disease models.

## Discussion

Unlike several other therapeutic areas such as haematology, cardiovascular, dermatology or HIV/AIDS, respiratory medicine faces a disappointingly low number of newly approved therapies [29]. This could be due to the inability to study organ-level complexities and pathological responses of human lung inflammation*.* To overcome this challenge, several disease models including in vivo and in vitro models have been proposed to recapitulate the feature of human lungs. However, animals cannot fully represent the human lungs since they have different anatomy, immune system, and inflammatory responses to the drugs when compared with human lungs [30]. Additionally, ethical and financial issues are raised in the use of animal models for testing drugs [31]. Therefore, an in vitro lung model could be an alternative option to replace and reduce the use of animal models in drug testing, screening, and validation.

However, current in vitro lung models have some limitations. They are either too simple and cannot recapitulate the important features in human lungs (*i.e.,* static lung models on transwells) or they are too complex but impossible to perform multimodal characterisation to validate the quality of the models (*i.e.,* lungs on a chip using microfluidics). Therefore, there is an urgent need to develop reliable and reproducible models that can mimic the complex human lung pathophysiology. Here, we demonstrated five key elements to develop the appropriate lung mimicking models: cell selection, membrane structure/constitution, environmental condition, cellular arrangement, and substrate/matrix composition.

Cell selection, environmental conditions, and cellular arrangements of the models are known to be critical to the success of the development of models. For the first time, we demonstrated the differences in the cell morphology, cellular barrier integrity, permeability, composition, and structure of multilayers of cells between the models to select the most appropriate one. Indeed, CNBio NHBE-NHLF (coated with laminin) exhibited comparable features as the commercial models such as continuous tight junctions, multiple layers of cells with fully ciliated cells and high electrical resistance value. Especially, the electrical resistance value CNBio NHBE-NHLF and commercial SmallAir™-HF healthy was in appropriate range for healthy in vitro lung models (from 700 to 1200 Ω·cm^2^) as indicated in previous study [21].

We for the first time determined the optimal membrane structure and extracellular matrix for the models using impedance measurement. Two common inserts (Corning and CellQart) and two common extracellular matrices (collagen type I and fibronectin) were used in our study. Impedance spectroscopy includes two components: electrical resistance (TEER) represents the barrier integrity of the models, and capacitance represents the growth and differentiation of the models. There were differences in the TEER value of the models grown on the Corning and CellQart inserts, regardless of the extracellular matrices. For the first 15 days of culture, the TEER value of the models grown on the Corning inserts was higher than the models grown on the CellQart inserts. Numerous groups found that a high TEER value is associated with well-developed differentiated epithelium ALI model [32, 33]. However, other groups found that the poorly differentiated cultured with the squamous structure have a greater TEER value than the differentiated, mucociliated structure [34, 35]. The lower TEER value of differentiated models in these studies represents for excessive active ion channels rather than improper cellular barrier [34]. Indeed, using AFM and nanoindentation, we for the first time observed that the CellQart inserts have a uniform and highly porous structure. This suggested that more nutrients are exchanged in the CellQart inserts and this event could interfere the TEER measurements. Therefore, the CellQart models expressed lower TEER value and this value do not represent the maturity of differentiated cultures. Indeed, cell capacitance of the models grown on the CellQart inserts increased from Day 13 to Day 24, which confirmed their growth and differentiation. Regarding the extracellular matrices, our result suggested that collagen promoted better growth and differentiation of the models than fibronectin. This is the first study using a quantitative method to accurately provide the insights into the model structure and development.

To optimise the disease models, we used the primary human lung cell line derived from COPD patients (DHBE). The damage of the cellular barrier integrity in our disease models was shown in the low electrical resistance value in comparison to the healthy models. In addition, the dynamic co-culture of DHBE and NHLF (CNBio DHBE-NHLF) had discontinuous tight junctions, which was consistent with the previous finding in in vitro asthma models [24, 36]. The cross-section histological images of this disease model exhibited overproduction and hypertrophy of goblet cells, and a reduction in the number of cilia. This result was consistent with the previous finding of excessive number of goblet cells and ciliary dysfunction occurred in COPD patients [37, 38]. Nevertheless, different features were shown in the control/commercial MucilAir™-HF COPD model, in which continuous tight junctions and no hypertrophy of goblet cells were presented in the MucilAir™-HF COPD. MucilAir™-HF COPD did not express critical pathological factors in COPD (discontinuous tight junctions and overproduction of goblet cells) as described in the developed model. Therefore, the developed disease models were likely to represent the pathological conditions in COPD patients.

The profile of cytokines secreted from healthy and diseased models was compared to understand the inflammatory response of each model. Generally, the expression level of all cytokines was elevated in disease CNBio DHBE-NHLF models. Importantly, the level of pro-inflammatory cytokines IP-10 and IL-6, critical regulators of lung inflammation [39, 40], were significantly higher in the disease models when compared to the healthy models. Increased level of IP-10 and IL-6 has been found in primary human alveolar and bronchial epithelial cells under the exposure to influenza viruses [41] – a frequent cause of exacerbations of COPD [42]. Therefore, IP-10 and IL-6 could be used as biomarkers for COPD developing models [43, 44]. Additionally, these cytokines can be employed as prediction tools for the recovery of damaged tissue after the treatment [45].

## Conclusions

In conclusion, our study emphasized the importance of five key technological and methodological components in the development of human pathophysiological mimicking lung models. In addition, the comprehensive characteristics of different models with varying complexity were revealed in this study, which will be useful for the researchers in the selection of suitable models for their specific applications. Importantly, we employed real-time and end-point quantitative and qualitative measurements to assess the physiological accuracy of the models. This is critical to improve the reliability and relevance of the data, thus facilitating the use of these models in the regulatory standards for new therapeutics for lung injury.

## Supplementary Information

**Additional file1:** **Table S1.** Summary of primary and secondary antibodies used in IF staining for sectioned models.

## Data Availability

All data that support the findings of this study are included within the article.

## Abbreviations

COPD: Chronic obstructive pulmonary disease
ALI: Air liquid interface
AFM: Atomic force microscopy
DMEM: Dulbecco's Modified Eagle Medium
QC: Quality control
NHBE: Normal bronchial/tracheal epithelial cells
DHBE: Diseased human bronchial/tracheal epithelial cells – COPD
CNBio: CNBio’s PhysioMimix™ 3D system
SmallAir™-HF healthy: Primary small airway epithelia cocultured with human airway fibroblast derived from healthy donors
MucilAir™-HF COPD: Primary epithelial cells cocultured with human airway fibroblast derived from COPD patients
TEER: Transepithelial electrical resistance measurement
H&E: Hematoxylin/eosin staining
IF: Immunofluorescence
SEM: Scanning electron microscopy
