# Supplementary material for: Advanced pathophysiology mimicking lung models for accelerated drug discovery
Source: Biomater Res. 2023 Apr 26;27:35. doi: 10.1186/s40824-023-00366-x (PMC10129441; doi:10.1186/s40824-023-00366-x)
Supplement: Supplementary file 1 — Additional file1: Table S1. Summary of primary and secondary antibodies used in IF staining for sectioned models. [file 40824_2023_366_MOESM1_ESM.docx]

**Table S1 Summary of primary and secondary antibodies used in IF staining for sectioned models**

| **View Side view** | **Primary antibodies** | **Secondary antibodies** |
| --- | --- | --- |
| Cross-section | Acetylated α-tubulin monoclonal antibody B512 conjugated to AF488 (clone: B-5-1-2, catalogue number: 322588, ThermoFisher Scientific) | |
|  | Anti-MUC5B antibody (rabbit) (polyclonal, catalogue number: HPA008246, Sigma Aldrich) | AF594 goat anti-rabbit IgG (catalogue number: A11012, ThermoFisher Scientific) |
|  | Anti-mucin gastric antibody (mouse) (monoclonal; clone: 45M1, catalogue number: M5293, Sigma Aldrich) | CF633 goat anti-mouse IgG (catalogue number: 20130-1, Assay Matrix Pty Ltd) |
| Apical side | Monoclonal ZO-1 (clone: ZO1-1A12, catalogue number: 339100, ThermoFisher) | CF633 goat anti-mouse IgG (catalogue number: 20130-1, Assay Matrix Pty Ltd) |
